# Correction to “ComBat‐Predict Enhances Generalizability of Neuroimaging Models to New Sites”

**DOI:** 10.1002/hbm.70625

**Published:** 2026-08-30

Xin, Y. , M. Gardner , N. J. Tustison , et al. 2026. “ComBat‐Predict Enhances Generalizability of Neuroimaging Models to New Sites.” Human Brain Mapping 47, no. 8: e70546. 10.1002/hbm.70546.42157534 PMC13581068

In the Acknowledgements section, the funding information was incomplete. The updated Acknowledgements section is as follows:

**Acknowledgements**

A.A.Chen’s work was partially supported by National Institutes of Health (NIH) Grant R01MH123550.

Y.X., A.B. and J.H.J. ’s work was supported in part by the National Institutes of Health/National Institute on Aging, under award number R01AG054159 (PI: Andreana Benitez).

The processing of the LBCC dataset was partially supported by NIH grants R01MH132934, R01MH133843, and R01MH134896. A complete list of funding sources that supported the collection and processing of data included in the LBCC can be found in Bethlehem et al, 2022.

We apologize for this error.

