# Supplementary material for: ComBat‐Predict Enhances Generalizability of Neuroimaging Models to New Sites
Source: Hum Brain Mapp. 2026 May 19;47(8):e70546. doi: 10.1002/hbm.70546 (PMC13581068; doi:10.1002/hbm.70546)
Supplement: Supplementary file 1 [file HBM-47-e70546-s001.docx]

# Supplementary

## *Age, Sex, and Diagnosis distribution in the ADNI dataset*

| Variable | N = 505^1^ |
| --- | --- |
| Age |  |
| Mean (SD) | 75.29 (6.71) |
| Median(Min, Max) | 76 (56, 90) |
| Sex |  |
| Male | 278 (55.0%) |
| Female | 227 (45.0%) |
| Diagnosis |  |
| Alzheimer's Disease | 115 (22.8%) |
| Low/Mild Cognitive Impairment | 239 (47.3%) |
| Cognitively Normal | 151 (29.9%) |
| ^1^ n (%) |  |

### **Table S1. Distribution of age, sex and diagnosis of the ADNI dataset**

## ***Sensitivity analyses: Linear Model Formulation***

As stated in results, examining the out-of-sample harmonization performance of CB-Predict under a linear formulation (**Figure 1** and **Figure S1.1**), we observed that for the ADNI subset used in the present study, comprising adults aged 55 and above, the linear formulation yielded consistently lower out-of-sample harmonization errors relative to in-sample harmonization. This may be attributable to the age-related trajectory of cortical thickness becoming approximately linear beyond a certain age threshold, such that a more flexible GAM formulation risks overfitting the covariate effect, thereby reducing the accuracy of out-of-sample predictions.

For the predictive modeling (**Figure 3**) and normative modeling (**Figure 4**) analyses, the linear formulation yielded comparable results to those obtained with the GAM-based approach, demonstrating that the main conclusions were robust to the choice of covariate model complexity (**Figures S1.2** and **S1.3**).

### **Figure S1.1. ComBat-Predict with linear formulation effectively harmonizes new sites, evaluated by leaving one site out**

The differences between the in and out-of-sample harmonization were low in for all 1-63 sites (site 64 is used as a reference site and thus excluded). **a)** The root mean squared errors (RMSEs) are low across different sample sizes in the testing sites, ranging from 3 to 20. Asterisks denote significant differences between in-sample and out-of-sample harmonized cortical thickness values (paired Wilcoxon signed-rank test with Benjamini-Hochberg correction): * ≥1 region, ** >20 regions, *** >40 regions (out of 62 total regions). **b)** The RMSEs were low across different proportions of Alzheimer's disease diagnosis in the re-ordered testing sites.

### **Figure S1.2. Comparison of cortical thickness centile scores across 62 regions from the reference LBCC controls and the ADNI test cohort (stratified by diagnosis: CN, LMCI, AD).**

(a) Centile scores derived from unharmonized ADNI cortical thickness data applied to the LBCC-trained normative model. (b) Centile scores derived after harmonizing the ADNI data using CB-Predict (**linear formulation**) based on the LBCC ComBat fit, followed by application of the LBCC-trained normative model.

### **Figure S1.3. Harmonization with CB-Predict with linear formulation reduces heterogeneity between healthy reference and test cohorts.**

Rank biserial correlation (RBC) effect sizes are shown for the comparison of centile score distributions between healthy LBCC controls and healthy ADNI controls across 62 cortical regions. Blue points represent effect sizes calculated using CB-Predict harmonized ADNI data, while yellow points represent effect sizes calculated using unharmonized ADNI data. Values closer to zero indicate greater similarity between the distributions of the two healthy control groups.

## ***NeuroHarmony Implementation and Evaluation***

As stated in **Results**, to compare CB-Predict with NeuroHarmony, we adapt the NeuroHarmony framework (Garcia-Dias et al., 2020) to our ADNI dataset and evaluate its performance with the same leave-one-site-out cross-validation approach applied to CB-Predict. For each held-out site *i*, we first apply ComBat harmonization to all remaining sites (training data) using a GAM formulation with covariates for age, sex, and diagnosis. We then calculate the site-specific “harmonization adjustments” for each cortical thickness measure as the difference between observed and harmonized values in the training data. Following the NeuroHarmony approach, we train separate random forest models (500 trees) for each of the 62 cortical regions, using age, sex, and diagnosis as predictors of the harmonization adjustments. These trained models are then applied to the held-out site *i* to predict the ComBat-based harmonization adjustments. Out-of-sample harmonized values for site *i* are obtained by adding the harmonization adjustments to the original cortical thickness measurements.

Consistent with our CB-Predict evaluation in Figure 1, we evaluate its performance by comparing these out-of-sample harmonized values to the reference in-sample harmonization obtained by including site *i* in the ComBat model. As demonstrated in **Figure S2**, NeuroHarmony produced higher out-of-sample harmonization RMSEs compared to CB-Predict.

### **Figure S2. Comparison of out-of-sample harmonization performance between CB-Predict and NeuroHarmony.**

Root mean squared errors (RMSEs) between out-of-sample harmonized and in-sample ComBat-GAM harmonized cortical thickness values, shown for each of the 63 evaluated sites ordered by sample size. Each boxplot summarizes the distribution of RMSEs across 62 cortical regions for a given site. Green boxplots represent CB-Predict; grey boxplots represent the adapted NeuroHarmony method.

## ***Sensitivity of CB-Predict harmonization to covariate effect shift and novel-site sample size***

CB-Predict, like standard ComBat, assumes a homogeneous covariate effect across sites when estimating site effects in new samples. This assumption may be violated when the covariate distribution of the novel site differs substantially from that of the training data. To evaluate the robustness of CB-Predict to such violations, we conducted a sensitivity analysis using simulated data with systematically varied age effects and novel-site sample sizes.

### *Simulation Setup and Evaluation*

We generate synthetic datasets consisting of 10 reference sites (in-sample) and one novel target site (out-of-sample). Each reference site is assigned a fixed sample size of n=20, while the sample size of the novel site (m) was varied from 5 to 100. Subject age and sex are bootstrapped from the empirical ADNI dataset to mimic realistic biological variability, and site-specific additive and multiplicative effects are sampled from the distributions observed in the real data. For consistency with the linear age effect in our simulations, we applied ComBat with a linear formulation, with age and sex included as parametric terms. Harmonization performance is quantified as the Root Mean Squared Error (RMSE) between in-sample harmonized values (obtained using standard ComBat on the pooled data) and out-of-sample harmonized values (obtained using CB-Predict), averaged across 62 cortical regions. This procedure is repeated for 500 iterations.

### *Impact of Sample Size*

As presented in Figure S1, increasing the sample size of the novel site was associated with a marginal increase in mean RMSE under a constant age effect. This likely reflects the fact that as the novel site grows larger, CB-Predict relies on a proportionally smaller training sample, modestly reducing the accuracy of out-of-sample site effect estimation relative to the standard ComBat reference baseline, which pools all sites jointly. When the age effect remains unchanged from training to testing, the median of mean RMSEs for out-of-sample harmonization remained lower than 0.03mm, when the sample size increased from 5 to 100.

### *Impact of Covariate Shift*

For a fixed sample size, CB-Predict demonstrated high robustness to shifts in the age effect between training and test samples. RMSEs remained visibly low even when the age effect increased by up to approximately twice the average effect (adding an additional 0.030 mm/year to the original mean decrease of 0.023 mm/year) observed in the reference data. A notable increase in harmonization error emerged only when the shift exceeded this threshold, at which point the association between large er novel-site sample sizes and increased RMSE also became more pronounced. Overall, these results suggest that CB-Predict is robust to realistic between-site differences in covariate effects, though caution is warranted when applying the method to samples with markedly atypical biological profiles relative to the training data.

### **Figure S3. Mean RSMEs between harmonized cortical thicknesses by CB-Predict and by ComBat/d-ComBat**

Mean root mean squared errors (RMSEs) between in-sample (ComBat) and out-of-sample (CB-Predict) harmonized cortical thickness values across 62 regions, shown for 500 simulated iterations per scenario. Each panel corresponds to a different age slope shift scenario, representing the deviation of the novel site's age effect from the reference effect estimated across 10 training sites (n=20 each). Colors indicate the sample size of the novel site (m), ranging from 5 to 100. Boxplots summarize the distribution of mean RMSEs across iterations for each sample size, with grey points denoting outliers.

## ***Diagnostic Classification Using Centile Scores***

To explore the potential of CB-Predict harmonization in diagnostic classification, we train random forest classifiers to distinguish between cognitively normal (CN) and Alzheimer's disease (AD) groups using the centile scores produced in Figure 3 as features.

Based on the subset of ADNI data with centile scores derived in Figure 3, we perform a 10-fold cross-validation by randomly partitioning the 30 ADNI sites into 10 folds of 3 sites each. In each fold, data from 27 sites are used for training and the remaining 3 held-out sites are used for testing. This process is repeated once for each of the 10 folds. For each train-test split, we train a random forest classifier (500 trees) on 62 regions of harmonized centile scores from the training sites (excluding age and sex as direct features since these are already accounted for in the centile score computation). The trained model is then applied to predict diagnostic probabilities for subjects in the held-out test sites, using both harmonized and unharmonized centile scores. Classification performance is evaluated using the area under the receiver operating characteristic curve (AUC).

For stability of results, this process of 10-fold cross validation is repeated using 10 different random seeds for the folds assignment. We calculate the mean AUC across the 10-fold cross-validation for each of the 10 random seeds and use a two-sided Paired Wilcoxon Signed-Rank Test to compare the 10 pairs of mean AUCs.

The mean AUCs were significantly higher when predicting with harmonized data (0.85± 0.02) compared to with unharmonized data (0.77 ± 0.04, *p* = 0.004). This result demonstrated that when applying an established brain chart to an unseen site, centile scores derived from harmonized data have greater discriminative ability for distinguishing Alzheimer’s disease from normal aging.

### **Figure S4. Comparison of Diagnostic Classification with Harmonized vs. Unharmonized Centile Scores**

Boxes represent the distribution of mean AUCs from the 10 random seeds (n=10 per box); each mean AUC was averaged across 10-fold Random Forest models.
